# Genome-wide cell-free DNA screening: a focus on copy-number variants

**DOI:** 10.1038/s41436-021-01227-5

**Published:** 2021-06-21

**Authors:** Jill Rafalko, Erica Soster, Samantha Caldwell, Eyad Almasri, Thomas Westover, Vivian Weinblatt, Philip Cacheris

**Affiliations:** 1 Laboratory Corporation of America, La Jolla, CA USA; 2 Department of Maternal Fetal Medicine and Perinatal Genetics, Capital Health System, Trenton, NJ USA

## Abstract

**Purpose:**

Of 86,902 prenatal genome-wide cell-free DNA (cfDNA) screening tests, 4,121 were positive for a chromosome abnormality. This study examines 490 cases screen-positive for one or more subchromosomal copy-number variants (CNV) from genome-wide cfDNA screening.

**Methods:**

Cases positive for one or more subchromosomal CNV from genome-wide cfDNA screening and diagnostic outcomes were compiled. Diagnostic testing trends were analyzed, positive predictive values (PPVs) were calculated, and the type of chromosomal abnormalities ultimately confirmed by diagnostic testing were described.

**Results:**

CNVs were identified in 0.56% of screened specimens. Of the 490 cases screen-positive for one or more CNV, diagnostic outcomes were available for 244 cases (50%). The overall PPV among the cases with diagnostic outcomes was 74.2% (95% CI: 68.1–79.5%) and 71.8% (95% CI: 65.5–77.4%) for “fetal-only” events. Overall, isolated CNVs showed a lower PPV of 61.0% (95% CI: 52.5–68.8%) compared to complex CNVs at 93.9% (95% CI: 86.6–97.5%). Isolated deletions/duplications and unbalanced structural rearrangements were the most common diagnostic outcomes when isolated and complex CNVs were identified by cfDNA screening, respectively.

**Conclusion:**

Genome-wide cfDNA screening identifies chromosomal abnormalities beyond the scope of traditional cfDNA screening, and the overall PPV associated with subchromosomal CNVs in cases with diagnostic outcomes was >70%.

## INTRODUCTION

In 2015, a large study using data from the California Prenatal Screening Program^1^ found that traditional cell-free DNA (cfDNA) screening (for common aneuploidies and sex chromosome abnormalities) had the capability to detect approximately 70–80% of the karyotypic abnormalities identified in fetuses and infants. The remaining 20–30% of abnormalities missed by traditional cfDNA screening included clinically relevant findings such as subchromosomal deletions/duplications, rare autosomal aneuploidies, and polyploidy, among others, and represented an area for growth of prenatal screening tests.

Genome-wide cfDNA screening was developed to expand the abnormalities detectable by prenatal screening. This testing became clinically available in the United States in 2015, and, as described in this study, has been performed in over 85,000 pregnancies since that time.

Despite years of experience with this testing, professional societies have yet to support genome-wide cfDNA screening in the prenatal setting, citing the need for additional data.^2,3^ A recent study from Soster et al. provided a review of the first three years of clinical experience with genome-wide cfDNA screening, including information regarding the sensitivity and specificity of the screening assay for the various aspects of the test, including copy-number variants (CNVs).^4^ The current study focuses specifically on a subset of abnormalities uniquely identifiable by genome-wide cfDNA screening: CNVs (i.e., subchromosomal gains or losses of material). Testing indications are examined for 490 cases that were screen-positive for one or more CNVs from genome-wide cfDNA screening, and outcome data are provided and analyzed for 50% (*n* = 244) of these cases.

## MATERIALS AND METHODS

Maternal blood samples submitted to Sequenom Laboratories® for MaterniT® GENOME testing were subjected to DNA extraction, library preparation, and genome-wide massively parallel sequencing as previously described by Jensen et al.^5^ Sequencing data were analyzed using a novel algorithm to detect aneuploidies and other subchromosomal events as described by Lefkowitz et al.^6^

Starting in 2015, samples from 86,902 consecutive, clinical genome-wide cfDNA screening specimens from singleton gestations were reviewed, and those positive for a subchromosomal CNV were compiled. In the context of this testing, a reportable CNV is defined as a gain or loss of chromosomal material 7 Mb in size or larger. In certain circumstances, CNVs below the 7 Mb threshold are reported when they are found in conjunction with an abnormality greater than 7 Mb in size that may be suggestive of an unbalanced structural rearrangement. Additionally, a select set of microdeletion regions (1p, 4p, 5p, 8q, 11q, 15q, and 22q) are included in this genome-wide cfDNA analysis, and abnormalities below 7 Mb in size are reported when the CNV involves these regions. However, the current study excludes from analysis isolated CNVs in these microdeletion regions.

Only samples from this cohort with diagnostic outcomes were included in positive predictive value (PPV) analysis. Diagnostic outcomes were obtained from two sources. First, outcome information was collected, when available, from the ordering provider. Second, positive cfDNA samples were cross-referenced with cytogenetic and single-nucleotide polymorphism (SNP) microarray diagnostic results submitted to Labcorp from chorionic villus sampling (CVS), amniocentesis, postnatal peripheral blood, and products of conception (POC) specimens during a corresponding timeframe. The process of consolidation and comparison of data across the three data sets (cfDNA results, cytogenetic results, and microarray results) was approved by Aspire IRB under clinical protocol SCMM-RND-402.

For a cfDNA sample to be considered a match to a cytogenetic and/or microarray specimen, the diagnostic and screening results were required to have identical patient identifiers (name and date of birth), and the collection date for the diagnostic test had to be within 90 days of the patient’s cfDNA screening date. When multiple diagnostic results (e.g., cytogenetic and microarray results, or CVS and amniocentesis results) were available for the same patient, results were combined under one final characterization.

A cfDNA result was classified as a true positive when one or more of the abnormalities identified by cfDNA screening were confirmed by karyotype or microarray analysis from diagnostic testing. A false positive classification was assigned when the abnormal screening result was not confirmed by diagnostic testing. PPVs were calculated by dividing the number of true positive results in a particular cohort by the total screen-positive results (true positives plus false positives) in that cohort.

Confidence intervals were calculated using the VassarStats Website for Statistical Computation. Comparison of ratios was performed using a two-sample, two-sided proportional *Z* test. Average CNV sizes were compared using a two-sided *t*-test for samples with unequal variances. For all calculations, *p* values less than 0.05 were considered statistically significant.^7^

## RESULTS

**Fig. 1:**
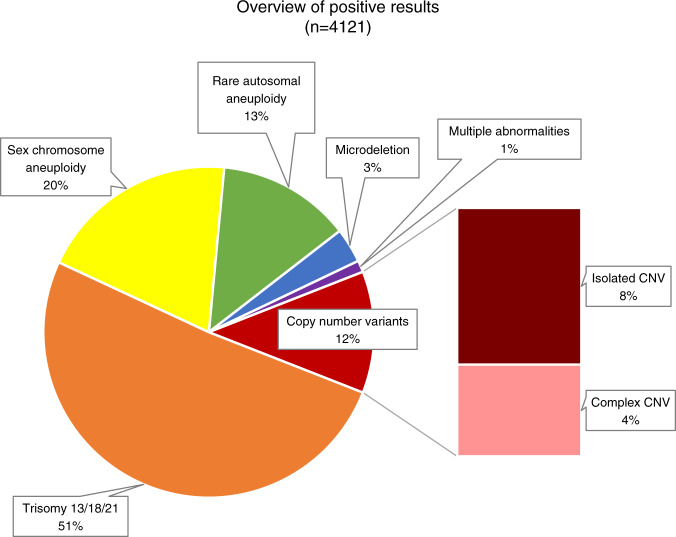
Screen positive cohort. Type of findings in the screen-positive cohort (*n* = 4,121).

Of 86,902 consecutive, clinical genome-wide cfDNA screening specimens, 4,121 positive results were issued, resulting in an overall positivity rate of 4.7%. Of these 4,121 cases, 11.9% (*n* = 490) were positive for one or more subchromosomal CNV, equating to an incidence of 1 in 177 (0.56%) for CNVs in the overall population screened. These cases were comprised of 309 isolated CNVs (i.e., one event identified in the genome) and 181 complex CNVs (two or more events identified across the genome), which would translate to an incidence of 1 in 281 (0.36%) for isolated CNVs and 1 in 480 (0.21%) for complex CNVs in the screening population. A breakdown of the type of findings in the overall screen-positive cohort can be seen in Fig. 1.

**Fig. 2:**
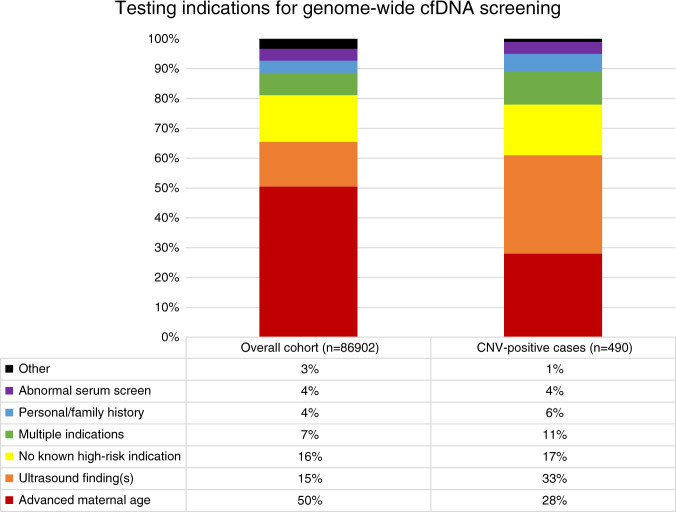
Testing indications. Testing indications for overall cases submitted for genome-wide cell-free DNA (cfDNA) screening compared to cases screen-positive for a subchromosomal copy-number variant (CNV).

The indication for cfDNA screening for the overall screening cohort (*n* = 86,902) compared to the CNV-positive cases (*n* = 490) can be seen in Fig. 2. Cases referred due to ultrasound findings as the sole indication for testing comprised 15% of the overall screening cohort, compared to 33% for the CNV-positive cohort. Furthermore, 41.6% (204/490) of CNV-positive cases had ultrasound findings as a reason for referral, either as the sole indication for testing, or in combination with other high-risk indications.

The 490 CNV-positive cases encompassed 675 unique, subchromosomal CNVs, ranging in size from 1.10 Mb to 207.60 Mb. These average deletion size was 23.04 Mb, the median was 16.30 Mb, and the interquartile range was 9.05–29.35 Mb. A summary of the CNV sizes for the screen-positive cases is provided in the Supplemental Information (Fig. S1).

Diagnostic outcome information was available for 50% (*n* = 244) of the 490 CNV-positive cases. When comparing cfDNA cases with diagnostic outcomes versus those without, some differences were observed. Significantly fewer patients with “no known high-risk indication” (*p* < 0.001) and significantly more patients with “ultrasound finding” (*p* = 0.018) or “personal/family history” (*p* = 0.049) had diagnostic outcomes. When cases with or without diagnostic testing were stratified by the presence or absence of ultrasound findings (either as the sole indication for cfDNA screening or in combination with other high-risk indications), significantly more cases with diagnostic testing had reported ultrasound abnormalities compared to cases without diagnostic testing (47% vs. 36%, *p* = 0.014).

For the overall cohort of isolated and complex CNVs with diagnostic outcomes, there were 181 true positive results and 63 false positive results, translating to a collective PPV of 74.2% (181/244; 95% CI: 68.1–79.5%) (Supplemental Information, Table S1). There were an additional 42 cases with no diagnostic testing performed, but “clinical correlation.” These were cases in which there was a known parental chromosome rearrangement and the fetus screened positive for one or more CNVs in the region of the parental abnormality. If these cases were considered in PPV analysis as true positives, the overall PPV would increase to 78.0% (223/286; 95% CI: 72.6–82.5%).

Of the 244 cases with diagnostic outcomes, 60% (*n* = 146) were cfDNA cases with isolated CNVs, and 40% (*n* = 98) were cases with complex CNVs. When outcomes were examined by the type of CNV identified, isolated CNVs showed a PPV of 61.0% (89/146; 95% CI: 52.5–68.8%), and complex CNVs showed a PPV of 93.9% (92/98; 95% CI: 86.6–97.5%) (Supplemental Information, Table S1).

Of the 89 true positive cases with isolated CNVs, 13 maternal events were identified. For these cases, fetal diagnostic testing was performed in 5 cases. The fetus was positive for the maternal CNV in 3 cases, negative for the maternal CNV in 2 cases, and fetal outcomes were unknown for the remaining 8 cases. Of the 92 true positive cases with complex CNVs, 4 maternal CNVs were identified. Fetal diagnostic testing was performed in two of these cases, and the maternal CNV was confirmed in one fetus, but not the other. Fetal testing was not pursued in the other two cases. This information can be used to calculate a PPV solely for fetal CNV events. Of the 234 total cases with fetal or neonatal testing, there were 168 true positive results, resulting in a “fetal-only” PPV of 71.8% (168/234; 95% CI: 65.5–77.4%).

The type of diagnostic testing, as well as the type of analysis performed on the diagnostic specimens, were analyzed for the cases with diagnostic outcomes (*n* = 244). A detailed review of this information can be seen in Supplemental Information (Fig. S2A and B).

## DISCUSSION

A review of the outcomes from genome-wide cfDNA cases positive for subchromosomal events can be divided into three parts: (1) analysis of test performance, (2) evaluation of the type of abnormalities ultimately identified in the true positive cohort, and (3) review of the potential reasons for discordant results.

### Test performance

As with any screening test, analysis of test performance is limited by the availability of outcomes from diagnostic testing. In the cohort of patients with CNV-positive results from genome-wide cfDNA screening, diagnostic results were available for 50% of these cases. A review of cases with and without diagnostic testing showed that patients referred for screening due to “ultrasound findings” or “personal/family history” were more likely to have diagnostic outcomes, whereas patients with “no known high-risk indication” were less likely to have diagnostic outcomes. It is important to remember that outcome data were obtained from provider feedback and matched diagnostic outcomes from internal databases. Therefore, outcome data may exist for additional patients; however, that information was not available for analysis in this study, making it difficult to draw conclusions regarding diagnostic testing trends in the study population. Furthermore, the lack of outcome data for half of positive cases is another limitation of the study as the population of patients that pursued diagnostic testing may have introduced bias and could have affected the calculated PPVs.

Focusing solely on cases with confirmed outcomes, the overall PPV for all CNVs was 74.2% (181/244; 95% CI: 68.1–79.5%) overall and 71.8% (168/234; 95% CI: 65.5–77.4%) for “fetal-only” cases (i.e., excluding maternal events). When analyzed by CNV type, cases positive for an isolated event showed a PPV of 61.0% (89/146; 95% CI: 52.5–68.8%), whereas cases positive for a complex event showed a PPV of 93.9% (92/98; 95% CI: 86.6–97.5%) (Supplemental Information, Table S1).

The overall PPV would increase to 78.0% (223/286; 95% CI: 72.6–82.5%) if cases with “clinical correlation” are considered. These include cases in which one or more fetal CNVs were identified and one of the parents carried an apparently balanced structural rearrangement involving that chromosomal region, but diagnostic testing was not performed. There were a significant number of additional cases in which the fetus was documented to have major structural abnormalities on ultrasound but no diagnostic testing was performed. Though many of these cases may be associated with the CNV identified by cfDNA screening, the authors chose not to include them in the “clinical correlation” group because of a lack of reasonable evidence to support a definitive association with the cfDNA finding.

### Type of abnormalities in true positive cohort

Of the 244 cases with diagnostic outcomes, the 181 CNV-positive cases that were confirmed by diagnostic testing showed a wide range of karyotype and microarray findings.

**Fig. 3:**
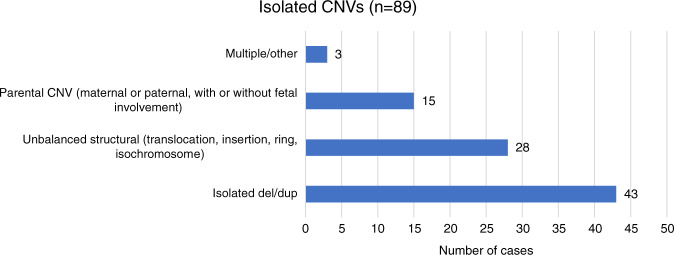
True positive isolated CNVs. Type of abnormalities identified by diagnostic testing in true positive cases for an isolated copy-number variant (CNV) from genome-wide cell-free DNA (cfDNA) screening (*n* = 89).

For the 89 cases that were confirmed to be true positive for an isolated CNV, 43 were confirmed to have a concordant, single deletion or duplication on diagnostic testing. Twenty-eight cases were found to be related to unbalanced structural rearrangements, including 13 unbalanced translocations, 11 isochromosomes, 3 ring chromosomes, and 1 insertion chromosome. For cases where two findings were detected by diagnostic testing, the size of the second finding was below the reporting threshold for the cfDNA assay, with many cases involving a CNV less than 1 Mb in size. There were 15 cases in which a parental CNV was involved: 13 maternal CNVs (3 in which the fetus was also found to carry the CNV, 2 in which the fetus was negative for the maternal CNV, 8 cases where diagnostic testing was not performed for the fetus) and 2 CNVs that were identified in the fetus that were paternally inherited. There were three additional cases with complex outcomes and multiple abnormalities (Fig. 3).

**Fig. 4:**
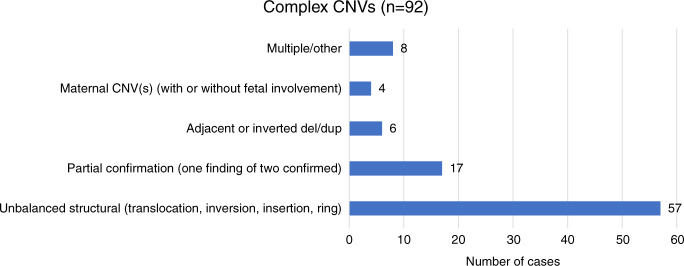
True positive complex CNVs. Type of abnormalities identified by diagnostic testing in true positive cases for a complex copy-number variant (CNV) from genome-wide cell-free DNA (cfDNA) screening (*n* = 92).

For the 92 complex cases (with two or more CNVs identified by cfDNA screening) that were confirmed to be true positive, 57 involved an unbalanced structural rearrangement on diagnostic testing, including 48 unbalanced translocations, 6 unbalanced inversions, 2 ring chromosomes, and 1 insertion chromosome. Six cases involved two findings (a deletion and a duplication) adjacent to one another. Four cases involved a maternal complex CNV: two cases in which the patient herself had an unbalanced translocation and corresponding phenotype, another case in which the patient had an inverted deletion/duplication of chromosome 5p, and one case in which the patient was diagnosed with a deletion of chromosome 22 and significant fibroids (which may account for the second finding, a mosaic deletion of chromosome 7q, an abnormality commonly documented in fibroid tissue^8^). Eight cases involved complex abnormalities, with some involving multiple structural abnormalities. The remaining 17 cases had two or more findings from cfDNA, with only one event confirmed (Fig. 4).

For cases in which only one of the two findings from noninvasive prenatal screening (NIPS) were confirmed (*n* = 17), the abnormality that was discordant showed sequencing data suggestive of mosaicism in several instances. These cases may involve a segmental “rescue” event in progress, such as telomere capture, which acts to stabilize an open deletion by acquiring material from another chromosome. This mechanism may result in mosaicism for the “stabilizing” CNV. One case for which there is evidence of this phenomenon involved a large (35.4 Mb) terminal duplication on 4p and a small (2 Mb) terminal deletion on 1p reported by cfDNA screening. The patient pursued amniocentesis, which confirmed only the 1p deletion. Placental studies were arranged after delivery and were consistent with a nonmosaic terminal 1p deletion and a mosaic (~51%) terminal 4p duplication.

A review of the specimen type submitted for diagnostic studies in the 17 cases where only one of the two cfDNA findings were confirmed showed 8 postnatal samples, 5 amniocentesis specimens, 2 POC, 1 CVS, and 1 test of unknown type. The placenta was not evaluated in at least 13 of these 17 cases; therefore, even if the two abnormalities detected by NIPS were truly present in the placenta (with subsequent rescue in the fetus), the testing ordered would not have been able to identify the second abnormality in 13 of these cases.

While chromosomal structure cannot be definitively discerned from cfDNA screening, cell-free DNA often shows characteristic sequencing data when certain abnormalities are ultimately identified by diagnostic testing. A review of typical data patterns for commonly encountered findings can be seen in Supplemental Information (Fig. S3). An unbalanced translocation often involves CNVs (gains or losses) involving the terminal segments of two different chromosomes (Fig. S3A). A recombinant chromosome, typically resulting from an unbalanced parental pericentric inversion, typically involves a terminal deletion on one chromosomal arm and a terminal duplication on the other arm (Fig. S3B). A supernumerary isochromosome is usually characterized by an entire chromosomal arm duplication, or duplication adjacent to the centromere (Fig. S3C). Ring chromosomes are often identified by two terminal deletions, one involving the p arm of the chromosome and the other involving the q arm of the same chromosome (Fig. S3D).

Genome-wide cfDNA screening using massively parallel sequencing may also identify CNVs that appear maternal in origin. As shown in Fig. S3E, a suspected fetal event shows a deviation from the normalized sequencing data generally consistent with the sample’s fetal fraction; whereas, a suspected maternal event shows a significantly larger deviation from the normalized sequencing data. This difference is related to the percentage of cfDNA contributed from the fetus alone versus the maternal cfDNA fraction. An algorithm is also applied to these results to determine the mosaicism ratio associated with the abnormal findings, which may help to identify which events could be fetal versus maternal in origin.^9,10^ Of note, when a maternal event is suspected from cfDNA sequencing data, an assessment of fetal status for that particular region is precluded. However, for cases in which a maternal event is present, each pregnancy (current and future) is at 50% risk to inherit the maternal CNV, which allows for prenatal diagnosis in these high-risk pregnancies, and for informed reproductive planning.

### Review of discordant results

As with traditional cfDNA screening,^2^ biological factors (e.g., mosaicism, vanishing twin, maternal condition) have the potential to confound the results of genome-wide cfDNA analysis and CNV detection. At a minimum, these biological limitations should be discussed in the event of a positive screening result, and may be appropriate to disclose during pretest counseling with the patient.

**Fig. 5:**
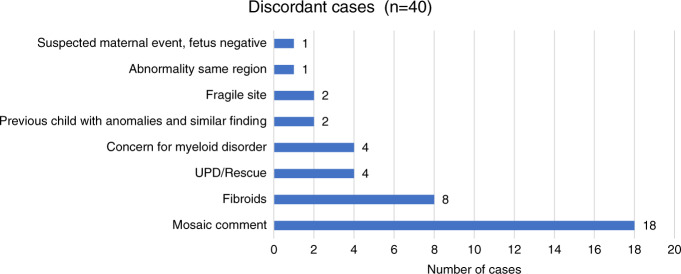
Possible explanations for discordant results. Potential biological reason for discordant finding from cell-free DNA (cfDNA) vs. diagnostic testing.

Of the 63 CNV-positive cases that were not confirmed by diagnostic testing, at least 40 cases (63%) had a probable biological explanation for the abnormal cfDNA result (Fig. 5). Of these cases, 34 were isolated CNVs and 6 involved complex CNVs.

Eighteen of the discordant cases included a “mosaic” comment on the laboratory report. This comment is derived from the patient’s sequencing data using the mosaicism ratio calculation that has been previously described.^9,10^ For these cases, the amount of abnormal cfDNA was low relative to the total fetal fraction of the specimen, suggesting that only a portion of the cfDNA contributed by the placenta may have been abnormal. A review of the testing performed in these cases found that 15 cases had diagnostic studies via amniocentesis, 2 cases had postnatal testing, and only one case had placental studies. Therefore, if CPM (Confined placental mosaicism) was present in any these 18 pregnancies, as suggested by the cfDNA results, only one of these cases had the potential to detect placental mosaicism based on the specimen submitted for diagnostic testing. Furthermore, it is possible for mosaicism to have been present at a higher level in the placenta, with cryptic or low-level mosaicism in the fetus, which escaped detection by diagnostic testing. In addition to placental mosaicism, some of these cases may have had discordant results due to a CNV “rescue” event, as discussed above, again reducing the likelihood of diagnostic confirmation.

At least eight of the isolated CNV cases that were discordant with diagnostic testing involved a pregnancy complicated by sizable or multiple fibroids. The genetic makeup of fibroids has been studied in the past and CNVs (both recurrent and novel) are commonly identified in these masses.^8^ As fibroids are known to shed cfDNA into maternal circulation,^11^ they provide a probable etiology for the abnormal cfDNA results in these cases.

Two of the discordant cases involved a 22-Mb mosaic 10q25.2 deletion in a known genomic fragile site, denoted FRA10B, and can be associated with acquired low-level maternal mosaicism for this deletion. This finding has been seen several times before from cfDNA screening, and has not been associated with clinical phenotype.^12^

Four cases involved CNVs that may be associated with myelodysplastic syndromes (i.e., del[5q], del[7q], and del[20q]), raising the possibility of an underlying, previously undiagnosed maternal condition.^13–15^ These cases were reported as positive for the CNV identified and the ordering clinician was contacted by a laboratory genetic counselor to discuss the potential etiology of these findings.

There were four cases with presumed rescue events in which the CNV was not confirmed by diagnostic studies (three via amniocentesis, one via postnatal microarray); however, segmental uniparental disomy (UPD) was present in the exact region of the predicted CNV.^16^ These cases provide evidence of a likely rescue event in the cell line that formed the fetus.

One case involved an 11.55-Mb duplication of chromosome 2q. Though this duplication was not confirmed, a 5.17-Mb deletion immediately adjacent to the abnormality predicted by cfDNA was confirmed by postnatal diagnostic testing. Similar to previous cases, there may have been a complex rescue event at play in this case.

In two cases, cfDNA screening identified an abnormality (or abnormalities) in the same region as a previously affected sibling to the fetus. One of these cases involved an 18p duplication and a 5p deletion (involving the cri-du-chat region) on cfDNA, but normal postnatal karyotype (46,XX). Interestingly, the proband’s sibling was reported to have previously been diagnosed with cri-du-chat syndrome. As 5p deletions in this region can be of variable size, it is possible that the finding may not have been detectable by karyotype in the newborn. A second, similar case, involved an isolated, 7-Mb deletion of 10q identified by cfDNA. Karyotype and microarray from amniocentesis were normal, with negative maternal cell contamination studies. The pregnant woman reportedly has another child with a 10q deletion in the same region. Though maternal karyotype was normal, there exists the possibility that the NIPS was detecting low-level maternal mosaicism of the 10q deletion, and the abnormality was missed by routine karyotyping (either due to the size of the abnormality or because of low-level or tissue-specific mosaicism).

The final discordant case involved a specimen with a strong mosaicism ratio, suggestive of a possible maternal CNV. The fetus was negative for the CNV, and though the report indicated a likely maternal event, maternal studies were not pursued.

As part of data analysis, CNV sizes for the 244 cases (346 total CNVs from both isolated and complex cases) with diagnostic outcomes were examined. A review of confirmed vs. discordant results for this overall cohort of 346 CNVs found that, in general, smaller CNVs were more likely to be confirmed than larger CNVs. The average size of CNVs that were confirmed by diagnostic testing (*n* = 251) was 19.08 Mb (median size 13.95 Mb) compared to an average of 33.36 Mb (median 25.50) for the CNVs that were discordant with diagnostic studies (*n* = 95). This difference was statistically significant (*p* < 0.0001). This trend (of confirmed findings being smaller, on average, than discordant findings) held true when isolated CNVs and complex CNVs were analyzed separately (*p* = 0.006 and *p* = 0.0007, respectively). Further details are provided in the Supplemental Information (Table S2).

Furthermore, for complex cases (with two or more CNVs identified) where only one finding was confirmed by diagnostic testing, the smaller of the findings confirmed more frequently than the larger finding. There were a total of 44 identified CNVs in this group, and the average size of confirmed CNVs was 11.80 Mb (median 11.68 Mb), which was significantly smaller than the average size of the discordant CNVs at 40.37 Mb (median 34.35 Mb), *p* = 0.0007. And in the 17 complex cases where both deletion and duplication were identified but only one CNV confirmed, the deletion was the confirmed CNV in 16 of these 17 cases. In the sole case where the duplication was confirmed but not the deletion, the SNP microarray studies detected a loss of heterozygosity in the same region as the deletion predicted by cfDNA screening, suggestive of a segmental rescue event.

Finally, it should be noted that not only the type of diagnostic test performed (e.g., CVS, amniocentesis, POC, postnatal), but also the type of analysis performed on the specimen (e.g., karyotype, SNP microarray, fluorescence in situ hybridization [FISH], UPD studies) are important factors to consider when determining whether diagnostic testing has truly ruled out a finding from cfDNA screening. For instance, a karyotype alone may not detect abnormalities on the smaller end of the reporting range for genome-wide cfDNA screening. Likewise, a karyotype does not have the ability to detect regions of homozygosity that could be indicative of a rescue event. Because a comprehensive or “ideal” diagnostic workup is not always possible in clinical practice, it should be considered as a potential limitation to confirmation of cfDNA findings.

### Closing thoughts

Genome-wide cfDNA screening offers patients and providers the opportunity to identify additional, clinically relevant chromosome abnormalities that would otherwise go undetected by traditional cfDNA screening. These abnormalities encompass a range of chromosomal findings historically limited to detection by karyotype, including isolated deletions/duplications and unbalanced structural abnormalities. This screening test is not a substitute for diagnostic testing. Patients who wish to maximize the detection of fetal chromosome abnormalities should be offered diagnostic testing with chromosomal microarray analysis.

The current study demonstrates that when a subchromosomal CNV is identified by genome-wide cfDNA screening, the observed PPV is high, at 74.2% overall and 71.8% for “fetal-only” events. The overall PPV appears higher for complex CNVs (93.9%) compared to isolated CNVs (61.0%), which may be related to the higher proportion of fibroid-related findings and potential segmental rescue events occurring in the case of isolated abnormalities.

In general, the average size of CNVs that were confirmed by diagnostic testing was smaller than the average size of CNVs that were discordant with results of diagnostic testing. This could be due, in part, to the fact that larger CNVs may be more poorly tolerated by the developing pregnancy than smaller abnormalities.

Despite the fact that genome-wide cfDNA screening was designed to mimic the resolution of a prenatal karyotype in the detection of unbalanced chromosome abnormalities, the findings from this study suggest that microarray may be a more suitable diagnostic test following the detection of a CNV by cfDNA screening. There were several instances where apparently isolated abnormalities from cfDNA screening were associated with an additional, often very small (<1 Mb) CNV when microarray studies were pursued. Additionally, microarray may be preferable, as some of the abnormalities on the lower end of the current cfDNA reporting range (~7 Mb) may not be detected by routine karyotype, depending on the size and location of the abnormality.

Finally, it is important to note that formal UPD studies may be indicated as one of the follow-up tests to a CNV-positive cfDNA result when a postzygotic rescue event is suspected. Segmental UPD may have implications for imprinted chromosomes or chromosomal regions, and could increase the risk for an autosomal recessive disorder for genes contained in the region of homozygosity.

In conclusion, data from over 86,000 screening samples have demonstrated that genome-wide cfDNA testing can provide patients with clinically relevant information beyond what is available from traditional prenatal screening tests. This study provides clinicians and professional societies with a large number of diagnostic outcomes from individuals with CNVs identified by cfDNA screening and adds to a growing body of evidence that supports the utility of this testing.

## Data Availability

Data that support the findings of this study are available upon request.

## Supplemental Information

Supplemental Information
