# Supplementary material for: Genome-wide cell-free DNA screening: a focus on copy-number variants
Source: Genet Med. 2021 Jun 21;23(10):1847–53. doi: 10.1038/s41436-021-01227-5 (PMC8486654; doi:10.1038/s41436-021-01227-5)
Supplement: Supplementary file 1 — Supplemental Information [file 41436_2021_1227_MOESM1_ESM.pdf]

Supplemental Information

Figure S1: Size distribution of the 675 CNVs identified in the 490 CNV-positive cases

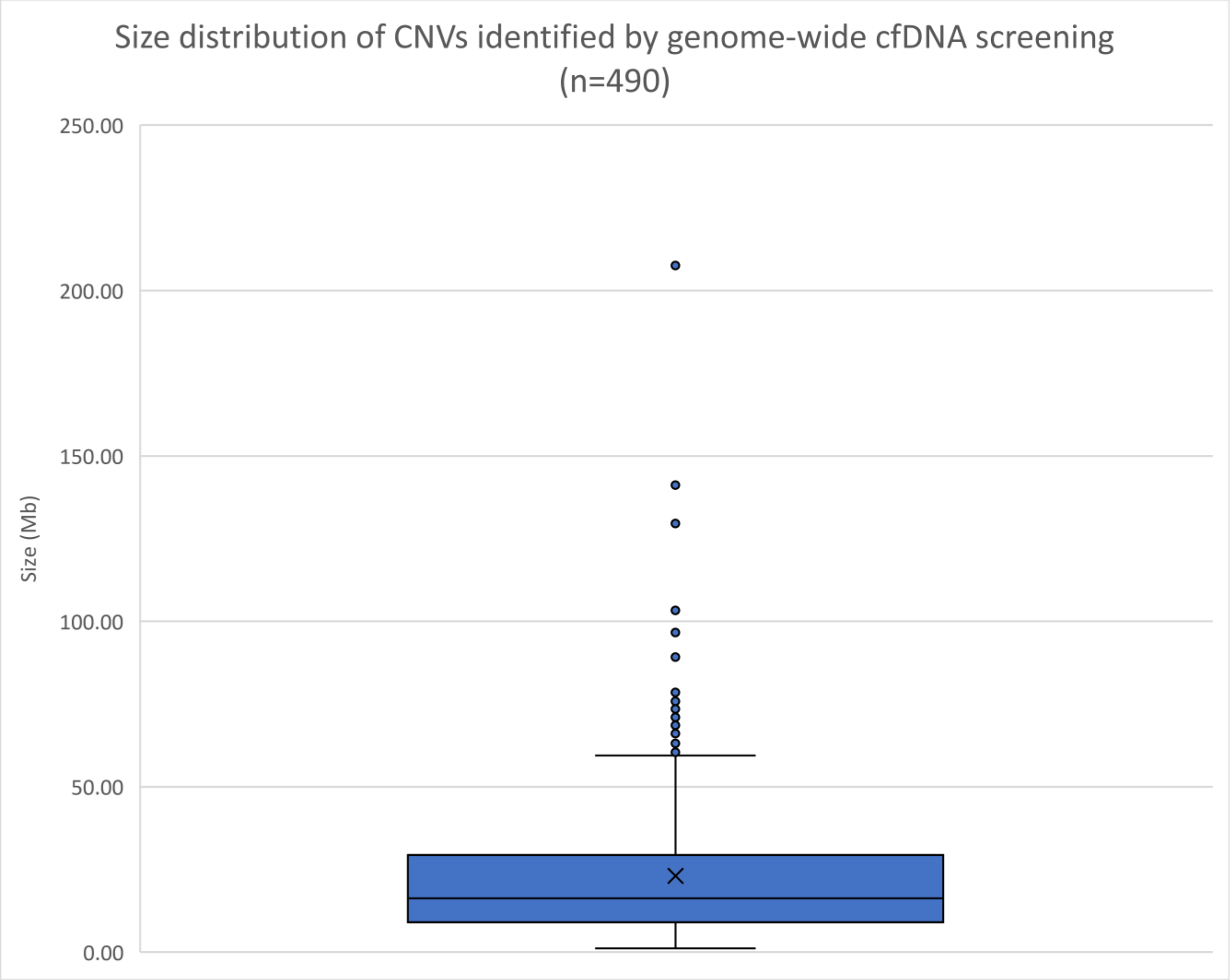

Mean: 23.04Mb
Median: 16.30Mb
IQR: 9.05 – 29.35Mb
Smallest CNV: 1.10Mb
Largest (subchromosomal) CNV: 207.60Mb

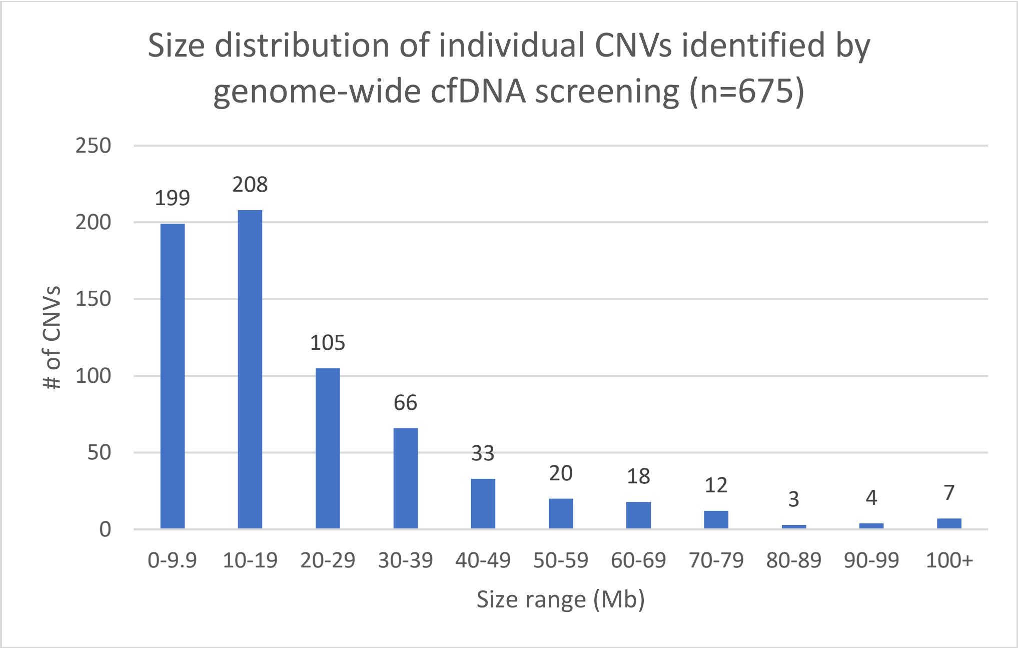

Size distribution of individual CNVs identified by genome-wide cfDNA screening (n=675)
# of CNVs
Size range (Mb)
0
50
100
150
200
250
0-9.9
10-19
20-29
30-39
40-49
50-59
60-69
70-79
80-89
90-99
100+
199
208
105
66
33
20
18
12
3
4
7

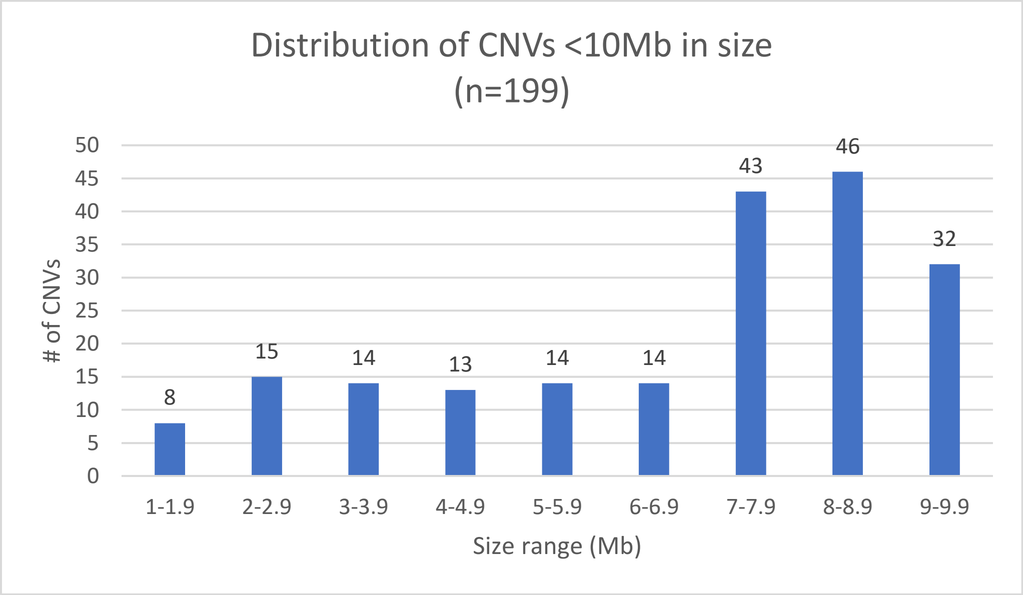

Distribution of CNVs <10Mb in size (n=199)
# of CNVs
Size range (Mb)
0
5
10
15
20
25
30
35
40
45
50
1-1.9
2-2.9
3-3.9
4-4.9
5-5.9
6-6.9
7-7.9
8-8.9
9-9.9
8
15
14
13
14
14
43
46
32

Figure S2: [A] Diagnostic specimen types submitted for analysis following a positive genome-wide cfDNA screen for a subchromosomal CNV (n=244)

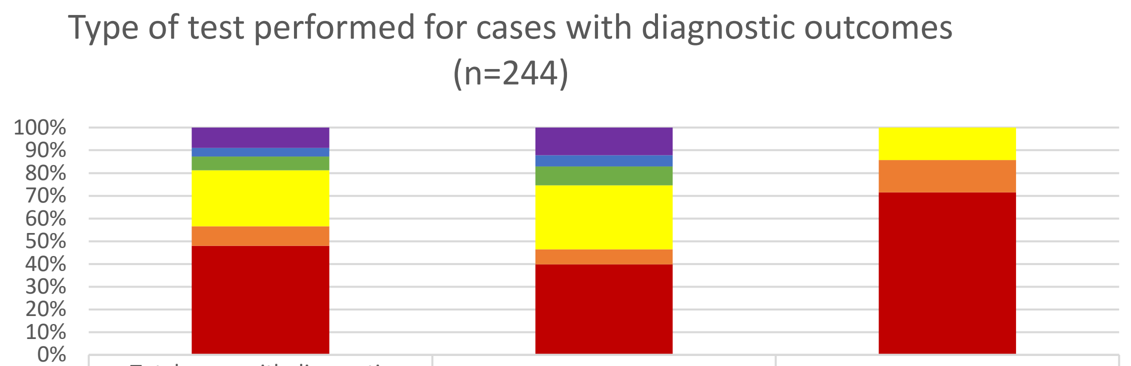

	Total cases with diagnostic testing (n=244)	Concordant (n=181)	Discordant (n=63)
Unspecified	22	22	0
Multiple	9	9	0
POC	15	15	0
Postnatal	60	51	9
CVS/placental	21	12	9
Amnio	117	72	45

[B] Type of analysis performed on diagnostic specimens submitted for testing following a positive genome-wide cfDNA screen for a subchromosomal CNV (n=244)

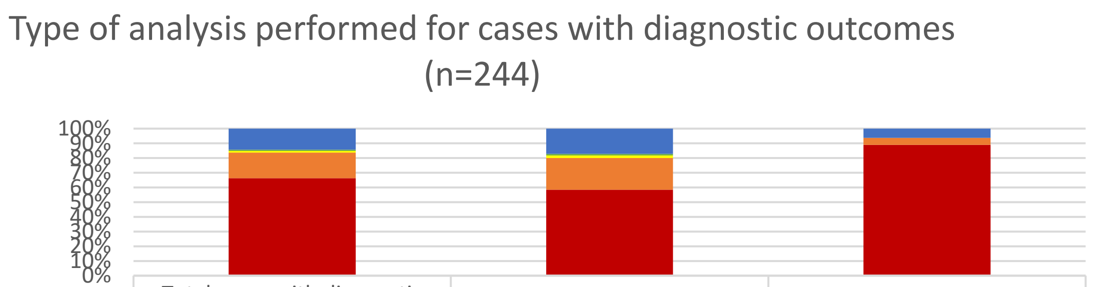

	Total cases with diagnostic testing (n=244)	Concordant (n=181)	Discordant (n=63)
Unspecified	35	31	4
FISH only	2	2	0
Karyo + FISH	3	3	0
Karyotype only	42	39	3
Array (+/- karyotype)	162	106	56

Note: FISH = FISH using probe for region of interest

Figure S3: Characteristic sequencing data for commonly identified chromosome abnormalities

(A) Unbalanced translocation – Example: translocation involving chromosomes 8 and 13

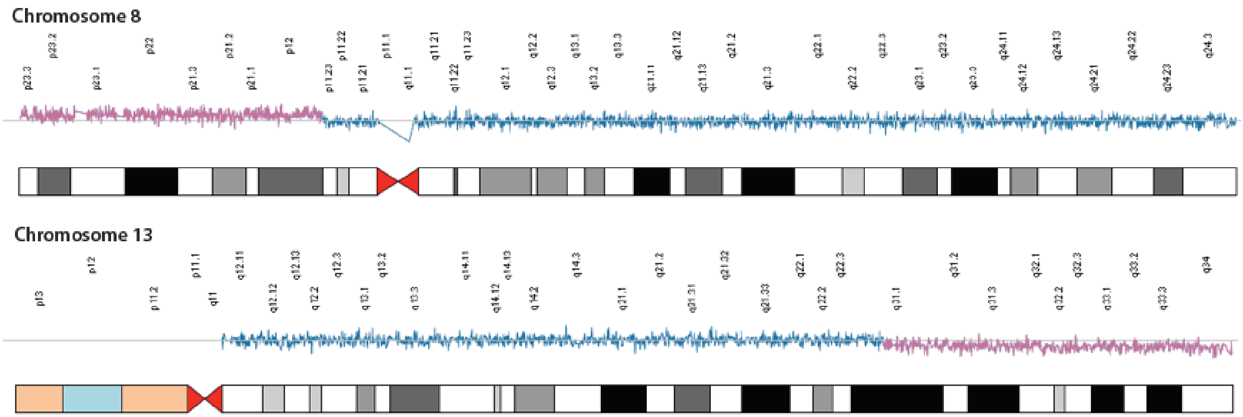

(B) Recombinant chromosome (unbalanced inversion) – Example: unbalanced product of a pericentric inversion of chromosome 1

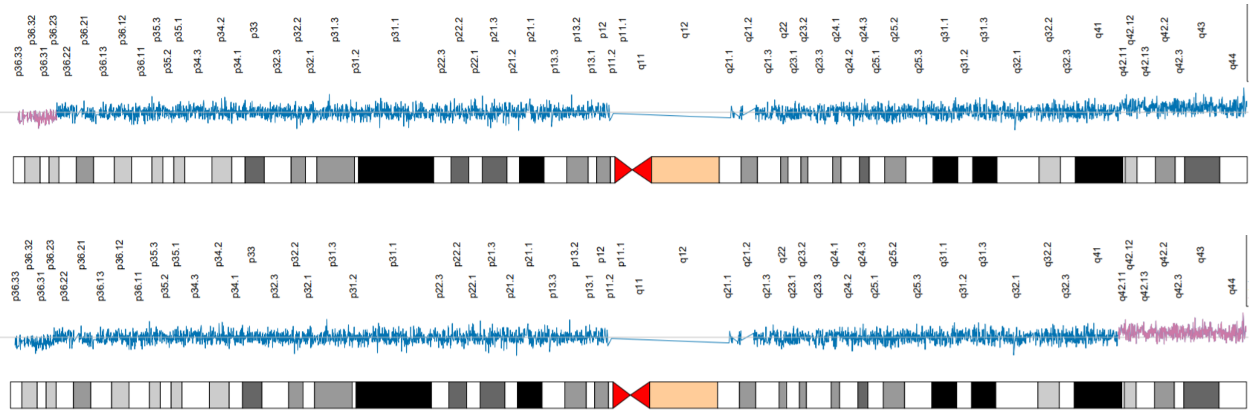

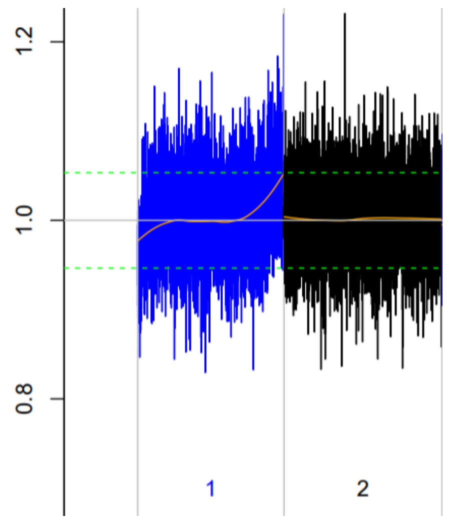

(C) Supernumerary isochromosome – Example: iso(18p)

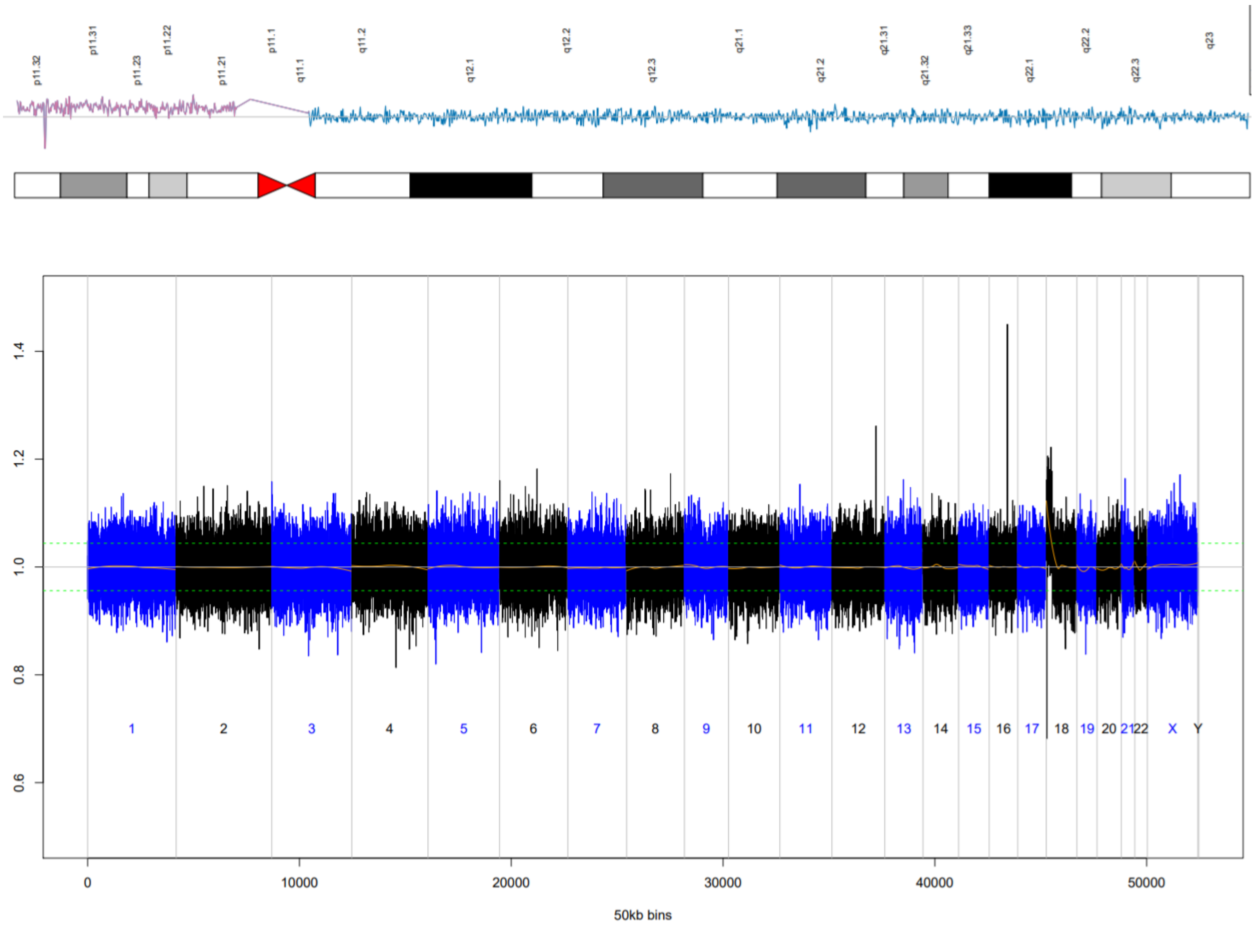

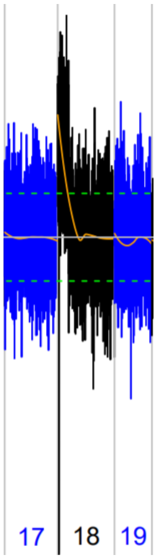

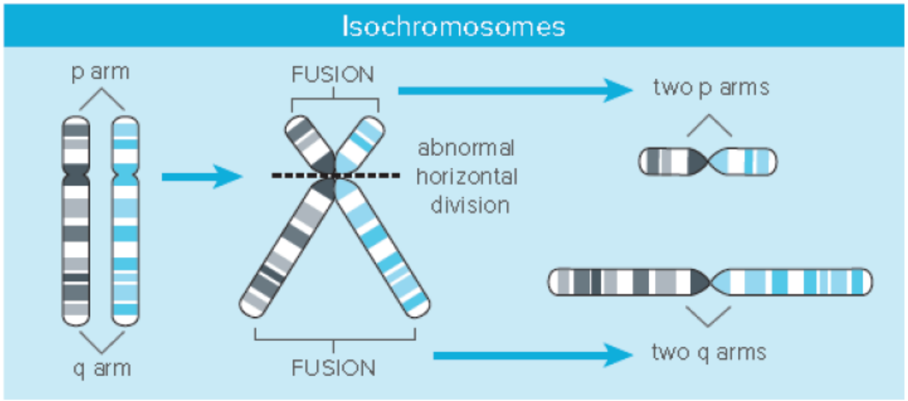

(D) Ring chromosome – Example: Ring 18

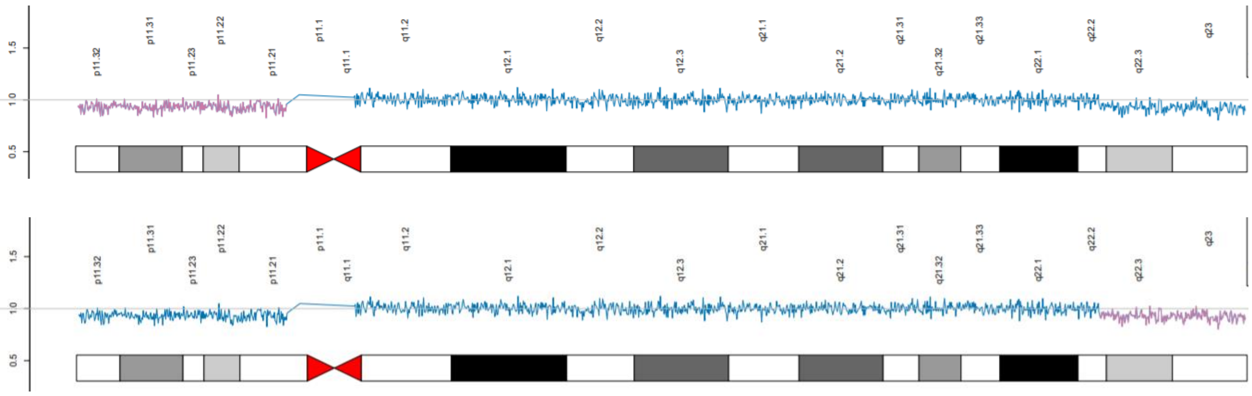

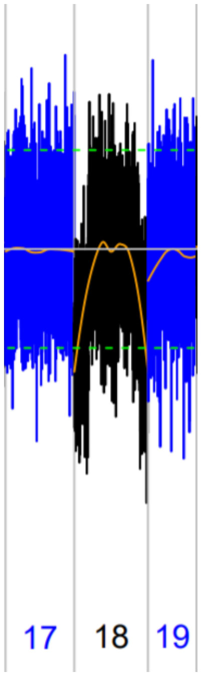

(E) Maternal versus fetal CNV – Example: 22q deletion

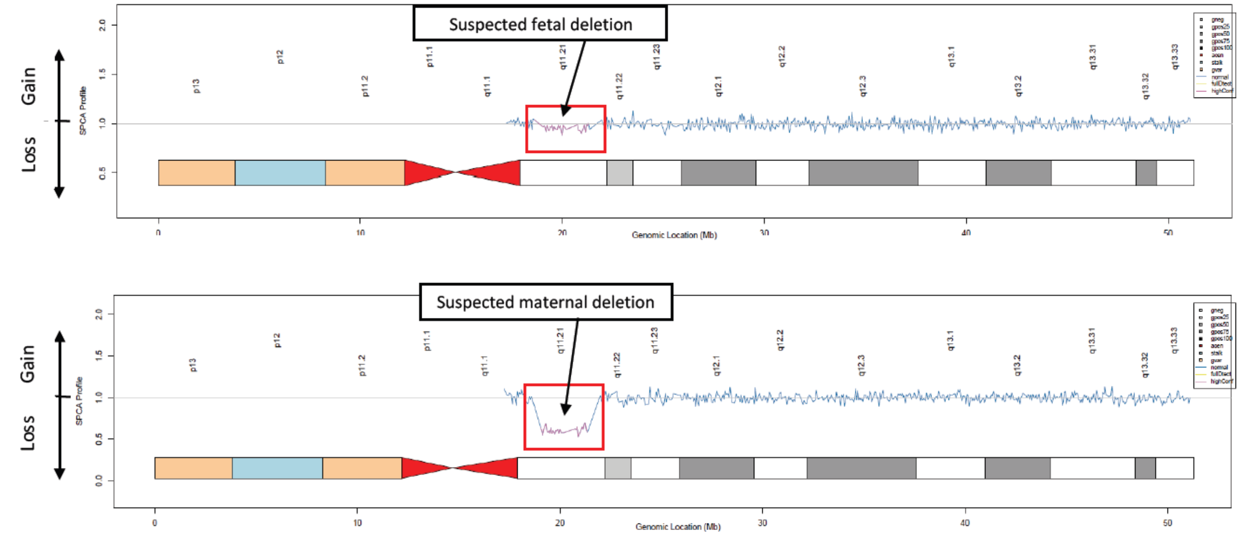

Table S1: Overview of outcome information for genome-wide cfDNA cases positive for at least one subchromosomal CNV.

	All CNVs	Isolated CNVs	Complex CNVs
**Total positive results**	490	309	181
**# with known diagnostic results**	244	146	98
**True positives**	181	89	92
**False positives**	63	57	6
**PPV**	74.2% [95% CI: 68.1-79.5%]	61.0% [95% CI: 52.5-68.8%]	93.9% [95% CI: 86.6-97.5%]

Table S2: Analysis of CNV sizes in cases with diagnostic outcomes

	Number of CNVs in cohort	Average size of confirmed CNVs in cohort	Average size of discordant CNVs in cohort	p-value
Isolated	146	21.50Mb (median: 14.80) n=89	30.54Mb (median: 22.50) n=57	p=.0064
Complex	200	17.75Mb (median: 13.20) n=162	37.60Mb (median: 29.68) n=38	p=.0007
All cases with diagnostic outcomes	346	19.08Mb (median: 13.95) n=251	33.36Mb (median: 25.50) n=95	p<.0001
